# TGF-β isoforms induce EMT independent migration of ovarian cancer cells

**DOI:** 10.1186/s12935-014-0072-1

**Published:** 2014-09-09

**Authors:** Jingfang Gao, Yihong Zhu, Mikael Nilsson, Karin Sundfeldt

**Affiliations:** Department of Obstetrics and Gynecology, Institute of Clinical Sciences, Sahlgrenska Academy at University of Gothenburg, Goteborg, SE-40530 Sweden; Sahlgrenska Cancer Center, Institute of Biomedicine, University of Gothenburg, Goteborg, SE-40530 Sweden

**Keywords:** TGF-β1, TGF-β2, TGF-β3, Epithelial ovarian cancer, Migration, Epithelial-to-mesenchymal transition, EMT, Cadherin, Snail, Zeb1

## Abstract

**Background:**

Transforming growth factor beta (TGF-β) plays major roles in tumorigenesis by regulating cell growth, epithelial-to-mesenchymal transition (EMT), migration/invasion and metastasis. The epithelial markers E-cadherin, claudin-3 and claudin-4, commonly decreased in human adenocarcinomas are actually up regulated during ovarian carcinogenesis. In human ovarian cancer TGF-β1 may either suppress or promote tumor progression, but whether other TGF-β isoforms (TGF-β2 and TGF-β3) exert similar effects is not known.

**Methods:**

In this study we investigated the ability of the TGF-β isoforms (TGF-β1-3) to induce proliferation and migration by BrdU labeling, scratch wound and trans-filter migration assays in the human serous adenocarcinoma cell-line NIH-OVCAR3. Transepithelial resistance was measured and EMT observed by light-microscopy. Expression of adherens-, tight-junction and EMT-related transcription factors was analyzed by qRT-PCR and immunoblotting.

**Results:**

All TGF-β isoforms dose-dependently inhibited NIH-OVCAR3 cell growth, stimulated tumor cell migration with similar efficiency. The mesenchymal marker N-cadherin and claudin-1 expression was induced and occludin down regulated. However, migrating cells retained an epithelial shape and E-cadherin expression. The E-cadherin repressor *SNAIL* mRNA levels remained low independently of TGF-β1-3 treatment while *ZEB1* expression was enhanced.

**Conclusions:**

TGF-β1, TGF-β2 and TGF-β3 promote migration of NIH-OVCAR3 ovarian cancer cells independently of cell proliferation and without conversion to a complete EMT phenotype. Epithelial ovarian cancer commonly metastasis to the surrounding tissue or inside the peritoneum rather than invading blood vessels to set distance metastasis. Our result raises the question whether ovarian cancer primarily spread via collective migration than via single cell invasion.

## Background

Epithelial derived epithelial ovarian cancer (EOC) accounts for more than 90% of all ovarian malignancies and is the most lethal gynecologic malignancy due to difficulties in diagnosing early stage disease [1]. More than 70% of EOC patients are diagnosed at an advanced stage with widespread dissemination in the peritoneal cavity. Therefore, increased understanding of molecular changes involved in ovarian cancer progression may lead to identification of novel targets for therapy.

Epithelial-to-mesenchymal transition (EMT) occurs in normal physiological processes essential for embryogenesis, tissue morphogenesis and wound healing but is also tightly linked to pathological conditions including fibrosis and cancer progression [2,3]. During EMT, epithelial cells typically lose their epithelial characteristics, including loss of cell polarity and cell-cell contact and acquire a spindle-shaped migrating phenotype. The key event of EMT is the switch of E-cadherin to N-cadherin, which renders the single cell more motile and invasive. Transforming growth-factor β (TGF-β) is a major inducer of EMT [2,3]. Several transcriptional factors including the zinc finger transcription factors Snail, Slug and Zeb play active roles conducting the EMT process [4]. TGF-β-induced EMT has been suggested to be associated with development and progression of EOC [5,6]. And our group have previously demonstrated that TGF-β induces typical EMT response in primary cultured human ovarian surface epithelium (OSE) accompanied by breakdown of epithelial barrier, down regulation of tight junction proteins claudin-1 and occludin and a switch from E-cadherin to N-cadherin expression [7]. The TGF-β1-induced morphological changes in OSE might be necessary to maintain the mixed epithelial-mesenchymal characteristics of native OSE and prevent inappropriate epithelialization of normal OSE, a proposed process for pre-neoplastic lesions in EOC development [8,9]. The outcome of TGF-β stimulation in OSE and EOC may thus be fundamentally different. EMT is far from well understood in relation to the development/progression/migration/invasion of epithelial ovarian cancer.

TGF-β exists as three isoforms, TGF-β1, TGF-β2 and TGF-β3 [10]. The three isoforms, TGF-β1-3 have more than 97% sequence identity in mammalian tissue and signal through activation of TGF-β receptors [11]. Clinical studies have provided evidence that the three isoforms are overexpressed and co-localized in ovarian cancers [11-13] and associated with advanced stage disease and reduced survival [12]. TGF-β1 has been described to induce EMT and an enhanced metastatic potential in OVCA429 ovarian cancer cells, a clear cell adenocarcinoma cell line [5,6]. However whether all three TGF-β isoforms are equally potent EMT inducers in all histologic subtypes of EOC has not been investigated.

In this study we investigated the ability of the TGF-β isoforms (TGF-β1-3) to induce proliferation and migration by BrdU labeling and scratch wound and trans-filter migration assays in the human serous adenocarcinoma cell-line NIH-OVCAR-3 cell-line. After TGF-β1-3 treatment EMT was assessed by quantitative changes in the expression of key molecules (N-cadherin, E-cadherin, occludin, claudins, Snail and ZEB) both at transcriptional and protein level. Morphologic changes of the cells were evaluated in light microscopy and transepithelial resistance was measured.

## Methods

### Cells, reagents and antibodies

The ovarian cancer cell-line NIH-OVCAR3 was purchased from American Type Culture Collection (ATCC, Manassas, VA) and cultured in a 1:1 mixture of M199/MCDB105 medium (Sigma Chemicals, St Louis, MO) supplemented with heat inactivated fetal bovine serum (FBS, Invitrogen Ltd, Paisley, UK) and penicillin-streptomycin (100 IU/ml-100 μg/ml; Life Technologies Ltd) in humidified atmosphere at 37°C. Recombinant human TGF-β1, TGF-β2 and TGF-β3 were purchased from R&D Systems (Abingdon, UK). For immunoblotting and real time PCR analysis, NIH-OVCAR3 cells were cultured at a density 6 × 10^4^/well in 12 wells plate until 60% confluent. After 24 h culture in 1% FBS, the cells were stimulated with TGF-β1, −2 and −3 in 1:1 mixture of M199/MCDB105 medium with 1% FBS for each experiment. Mouse monoclonal antibodies against N-cadherin and E-cadherin were obtained from BD Bioscience (San Jose, CA). Rabbit polyclonal against claudin 1 and mouse monoclonal against occludin were purchased from Zymed Laboratories (South San Francisco, CA). All research has been conducted in accordance with the declaration of Helsinki and the local ethical review board in Gothenburg.

### Cell proliferation – BrdU labeling

Cell proliferation was determined by measurement of BrdU incorporation during DNA synthesis using a colorimetric assay Cell Proliferation ELISA, BrdU kit (Roche Applied Science, Mannheim, Germany), according to the manufacturer’s instruction. NIH-OVCAR3 cells were cultured in a 96-well plate at a density 1 × 10^4^ cells/well in 100 μl culture medium at 37°C for 24 h and then treated with TGF-β1, TGF-β2 or TGF-β3 (1-10-50 ng/ml) in 100 μl culture medium with 1% FBS or 0% FBS for 72 h. 100 μM BrdU in labeling solution was added to the wells and the cells were incubated for additional 4 h at 37°C. Labeled cells were fixed and DNA was denatured by incubation in FixDenat (200 μl/well) for 30 min and thereafter, 100 μl/well anti-BrdU-POD working solution was added and incubated for 90 min. The cells were rinsed three times with PBS and incubated with 100 ml substrate solution containing tetramethyl-benzidine for 5 min and finally, 25 μl 1 M H_2_SO_4_ was added to each well for 1 min on a shaker at 300 rpm. Absorbance of the samples was measured using a spectrophotometric plate reader at 450 nm with a reference wavelength at 690 nm. All steps were performed in room temperature (RT). Culture medium without FBS was used as a control for nonspecific binding. Experiment was performed in triplicate wells and repeated four times.

### Cell migration - scratch-wound assay

The NIH-OVCAR3 cells were cultured in 6-well dishes (seeding density 1 × 10^6^cells/well). Confluent cell monolayers was disrupted by standardized wound scratching using a sterile 200 μl pipette tip and incubated in culture medium with 1% FBS without or with 10 ng/ml TGF-β1, TGF-β2 or TGF-β3 for 72 h. Migration of cells into the bare area and recovering of monolayer was evaluated every 12 h until 72 h by a phase contrast microscope and digitally photographed (Nikon Diaphot 300; Nikon, Tokyo, Japan).

### Cell-invasion filter assay

Filter transmigration of NIH-OVCAR3 cells was measured using Transwell® 6.5 mm insert (Costar®, Corning Incorporated, Corning, NY) and BD BioCoat^TM^ invasion chambers with (BD Biosciences, Bedford, MA) both with 8.0 μm pore size. The NIH-OVCAR3 cells (1 × 10^5^ cells per well in 300 μl) in culture medium with 0.1% BSA, were seeded in the upper chamber of the filter inserts and TGF-β1, TGF-β2 or TGF-β3 (10 ng/ml) enriched medium was added to the lower chamber. After incubation for 72 h at 37°C all cells that did not enter the filter were removed by gently scraping with a wet cotton swab on the upper side of the filter. Cells that migrated to the bottom filter surface were fixed by soaking insert in 4% formaldehyde for 2 min, stained with hematoxylin and eosin and air-dried. Filters cut out from inserts were mounted up side down on glass slides. Cells were counted under light microscope. The experiment was performed three times.

### Immunofluorescence

NIH-OVCAR3 cells were grown on Ø 19 mm glass cover slips (Histolab, Histolab Products AB, Gothenburg, Sweden) in a 12 wells plate until 60-70% confluence. Starvation with 1% FBS medium for 24 h, then stimulated with TGF-β1 10 ng/ml for 24 h, 48 h and 72 h. Cells were fixed in ice-cold methanol for 10 min and then rinsed briefly and stored in phosphate-buffered saline (PBS) two times. The cultured cells on coverslips were incubated with 1% BSA in PBS, followed by primary E-cadherin monoclonal antibody (1:1000) for 1 h at room temperature. Bound antibodies were visualized by ALEXA fluor secondary anti-mouse antibody (1:500, Molecular Probes, Eugene, Oregon, USA). The coverslip were mounted with the cells facing towards the microscope slide with a drop of vectashield mounting medium with DAPI (Vector Laboratories, Cambridgeshire, UK). The coverslips were sealed with rubber glue to prevent drying and movement under microscope.

### Immunoblotting

NIH-OVCAR3 cells were lysed using Mammalian Cell Lysis Kit (MCL1, Sigma-Aldrich, Saint Louis, Missouri, USA) and total protein content was estimated using the Micro BCA™ Protein Assay Kit (Pierce, Rockford, IL, USA). Twenty-five micrograms protein from each sample were boiled at 70°C for 10 min and loaded onto a SDS-polyacrylamide gel (NuPAGE® Novex 4-12% Bis-Tris Midi Gel, Invitrogen, Carlsbad, CA) along with Precision Plus Protein™ standards (Bio-Rad Laboratories, Hercules, CA). After electrophoresis, proteins were transferred to polyvinylidene fluoride membrane (Invitrogen, Carlsbad, CA) using a blotting system (iBlot™ Gel Transfer Device, Invitrogen, Carlsbad, CA) and incubated with primary antibody mouse monoclonal anti-N-cadherin (1:5000) and E-cadherin (1:5000) as well as rabbit polyclonal anti-claudin1 (1:800) and occludin (1:800) at 4°C overnight. In the next day, the membranes were washed with PBS and incubated with ECL™ peroxidase labeled second anti-mouse/rabbit antibody (1: 10000) (GE Healthcare, Bio-Science, Buckinghamshire, UK) in RT for 1 h, followed by Amersham™ ECL Advanced™ Western Blotting Detection Kit, (GE Healthcare, Bio-Science, Buckinghamshire, UK) according to manufactures instruction. The chemiluminescent signal was visualized by a LAS-4000 CCD camera (Fujifilm, Tokyo, Japan) allowing individual bands to be quantified by densitometry using the Quantity one software (Bio-Rad Laboratories). The loading was evaluated by staining the gels with Coomassie blue. Signal intensities of the individual protein were normalized to the gels stained with Coomassie blue and presented as ratios that represent arbitrary densitometric units (ADU) of relative abundance [14,15].

### Quantitative real time PCR

Total RNA was extracted from cultured NIH-OVCAR3 with or without TGF-β1-3 treatment for 0, 24 and 72 h using Qiagen Micro plus kit (Qiagen, Hilden, Germany) according to the manufacturer’s instruction. RNA concentrations were determined by Nanodrop ND-1000 (Thermo Fisher Scientific, Wilmington, DE, USA). Complementary DNA (cDNA) was synthesized from 1 μg total RNA using the High Capacity cDNA Reverse Transcription Kit (Applied Biosystems, Foster city, CA USA) according to the manufacturer’s protocol. Quantitative RT-PCR was carried out using TaqMan® Gene Expression Assays (Applied Biosystems) for each of the following genes with specific primers: E-cadherin (Hs00170423_m1), N-cadherin (Hs00983062_m1), occludin (Hs00170162_m1), claudin 1 (Hs00221623_m1), snail (Hs00195591_m1), and zeb1 (Hs01566407_m1) (Applied Biosystems). Large ribosomal protein (Hu RPLPO: 4333761 F) was used as an endogenous control. Each amplification reaction consisted of 10 ng cDNA, 1 × probe-mix and 1 × TaqMan® Gene Expression Master Mix to a final volume of 25 μl. After control for similar amplification efficiency of the target gene and endogenous control, relative expressions were calculated with the comparative Ct method (ΔΔCt). The mRNA expression of target genes was normalized to expression of the endogenous control. All samples were run in duplicate for both target and control genes and a mean of these values were used as a single observation in the presentation of data and in the statistical analysis.

### Transepithelial resistance (TER) measurements

Transepithelial resistance (TER) was measured by the Millicell Electrical Resistance System (Millipore Corp., Bedford, MA) with cells grown on Costar® Transwell® Permeable Support 0.4 μm Polyester Membrane 6.5 mm Insert (Cornig Incorporated, Corning, NY). NIH-OVCAR3 were seeded at a density 1 × 10^5^ cells per insert in 300 μl culture medium with 10% FBS in the Corning Transwell insert. When the cells reach confluent, the cells were treated with TGF-β1, TGF-β2 or TGF-β3 (10 ng/ml) in the same medium for up to further 96 h. Cells cultured without TGF-β were included as controls. TER was monitored at different time points after TGF-β addition and calculated by subtracting the background resistance of a blank filter that contained only medium and by multiplying the surface area of the filter membrane (0,33 cm^2^ for the 6.5 mm inserts) [7].

### Statistics

Statistical analyses were performed using STATISTICA 7.1 (StatSoft.com). Calculation of means and standard errors of the mean were performed with Excel Microsoft Office 2007. Statistical differences between treated cells and control groups regarding cell proliferation, cell migration, mRNA and protein expression were calculated using the Student’s test. *P* value less than 0.05 was considered as statistically significant.

## Results

### Inhibition of cell growth by TGF-β1, TGF-β2 and TGF-β3 in NIH-OVCAR3 cells

**Figure 1:**
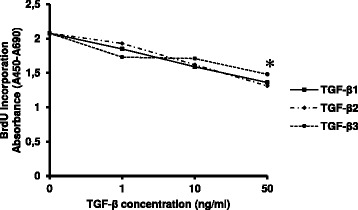
**TGF-β** **1, TGF-β** **2 or TGF-β** **3 inhibit NIH-OVCAR3 cell growth.** Cells were treated with any TGF-β isoforms at indicated concentrations for 72 h. Cell proliferation was determined by BrdU incorporation. Experiments were performed in quadruplicate; with the data represent the mean of the quadruplicate of each group ± SE. Values were compared with their respective control without TGF-β using Student’s *t*-test. *P < 0.05

To minimize the possibility that any effect on migration depend on altered proliferation rate, experiments were conducted in low serum. As expected from earlier published data, administration of TGF-β1 to NIH-OVCAR3 cells resulted in growth inhibition. NIH-OVCAR3 where also equally suppressed by TGF-β2 and TGF-β3 treatment (Figure 1). Addition of TGF-β1-3 at 1–10 ng/ml did not significantly alter BrdU-labeling whereas 50 ng/ml decreased tumor cell proliferation (p < 0.05). Importantly, similar inhibitory effect of TGF-β1-3 on cell growth was observed in cells cultured with and without FBS, since the purpose of this study was to elucidate the potential stimulatory effect of TGF-β isoforms on ovarian tumor cell migration and whether it is related to EMT. The OVCAR-3 ovarian cancer cell line express both TGF-β type I and II receptors [16].

### Effect of TGF-β isoforms on NIH-OVCAR3 morphology and cell migration

**Figure 2:**
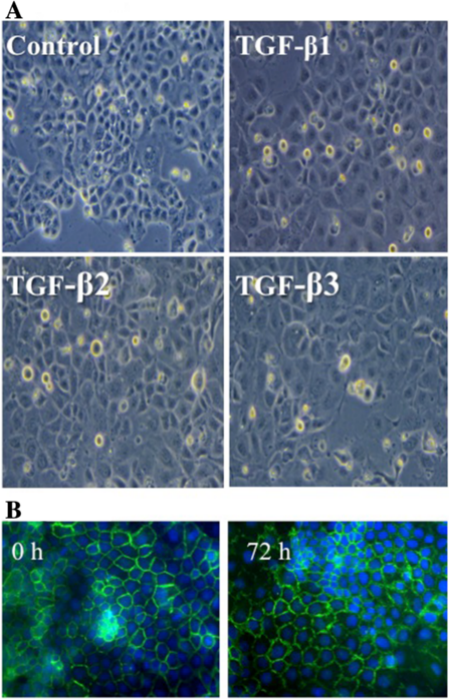
**Light micrograph and immunofluorescens pictures of cultured NIH-OVCAR3 cells treated with and without 10 ng/ml TGF-β** **1, TGF-β** **2 or TGF-β** **3 for 72 h. A.** There was no change of phenotype in any TGF-β isoform treated NIH-OVCAR3 cells compared with controls. **B.** Immunofluorescens staining of cultured TGF-β1 treated NIH-OVCAR3 after 0 h and 72 h. Cell-membrane bound E-cadherin was seen at both time points with and without TGF-β1.

NIH-OVCAR3 as well as normal ovarian epithelium is previously known to grow with typical cobblestone appearance, express E-cadherin and possess junctions typical of epithelial cells [7]. One hallmark of EMT is the phenotypic change in epithelial cell morphology as a response to TGF-β stimulation. Surprisingly, NIH-OVCAR3 cells displayed a cobblestone shape up to 5 days with TGF-β1, TGF-β2 or TGF-β3 treatment without signs of transition to a mesenchymal phenotype (Figure 2). This was evident in both the sub-confluent and confluent culture phase.

**Figure 3:**
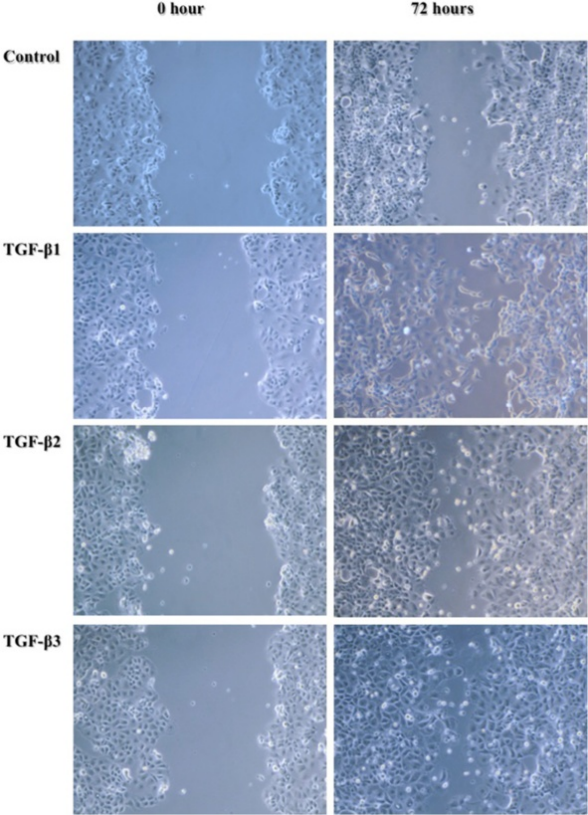
**Cell migration was evaluated by scratch wound assay.** Confluent cells were treated with 10 ng/ml TGF-β1, TGF-β2 or TGF-β3 in medium with 1% FBS for 72 h. Wound closure was monitored by light microscopy at 0 and 72 h.

NIH-OVCAR3 cells were grown to confluence for the assessment of TGF-β induced migration in a scratch wound assay in low serum conditions. In the absence of TGF-β most of the wounded area remained free of cells 72 h after injury (Figure 3). This indicated that NIH-OVCAR3 has a low spontaneous migrating capacity. However, TGF-β promoted wound closure by >50% in the same time period. All three TGF-β isoforms appeared to be equally potent. Interestingly, although migrating, NIH-OVCAR3 cells were slightly enlarged and the epithelial shape was not altered in response to TGF-β (Figure 3). Further, migration across permeable filter with and without matrix coating was investigated. Results showed that NIH-OVCAR3 cells were unable to cross the filter even when TGF-β was present in the opposite culture medium (data not shown).

### Effect of TGF-β isoforms on cadherin expression in NIH-OVCAR3

**Figure 4:**
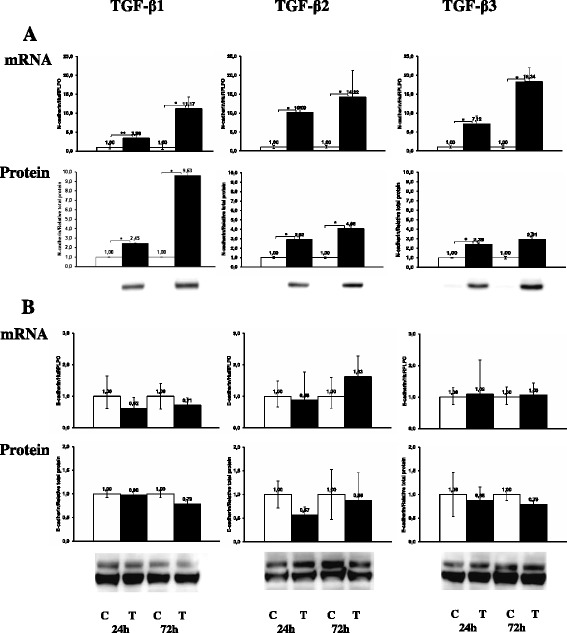
**Effect of TGF-β** **1, TGF-β** **2 or TGF-β** **3 on the mRNA and protein expression of adherens junction molecules. (A)** Significant increase of N-cadherin expression was observed in TGF-β treated NIH-OVCAR3 cells compared with controls whereas **(B)** there was no difference in the mRNA and protein expression of E-cadherin between TGF-β treated NIH-OVCAR3 cells and controls. *P < 0.05.

**Figure 5:**
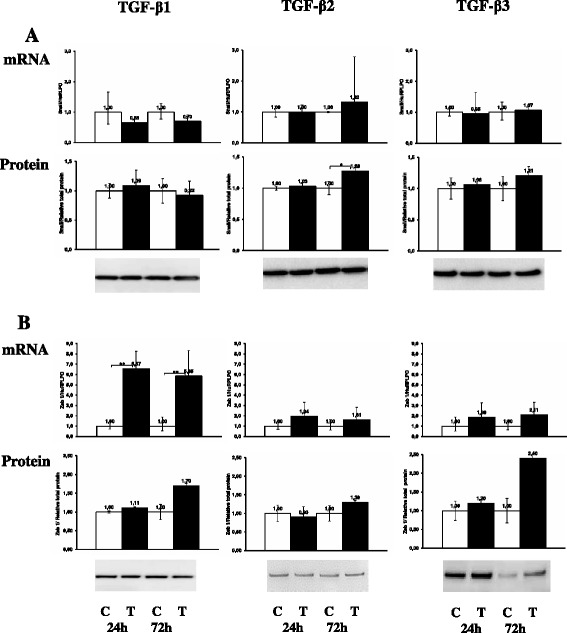
**Effect of TGF-β** **1, TGF-β** **2 or TGF-β** **3 on mRNA and protein expression of transcription factors.** There was **(A)** no difference in the mRNA expression of Snail between TGF-β treated NIH-OVCAR3 cells and controls, but **(B)** an increase of mRNA expression of Zeb 1 was observed in TGF-β treated NIH-OVCAR3 cells compared with controls. *P < 0.05.

Another hallmark of TGF-β induced EMT is down-regulation of E-cadherin and up-regulation of N-cadherin expression. As shown in Figure 4B, the expression of E-cadherin mRNA and protein was not affected by any TGF-β isoform treatment for up to 72 h. With immunoblotting we could detect a stronger and a weaker E-cadherin band corresponding to molecular weight of approximately 120 kDa and 135 kDa. They accounted for the precursor and processed E-cadherin species and were present regardless of treatment indicating that its turnover likely was unchanged. The cell membrane bound localization of E-cadherin was preserved and distinct after 72 h TGF-β stimulation (Figure 2b). N-cadherin was significantly (p < 0.05) increased by TGF-β at both mRNA and protein levels (Figure 4A). In fact, in untreated NIH-OVCAR3 cells N-cadherin was barely detectable. The increased expression was evident after 24 h and more pronounced after 72 h treatment in particular by TGF-β1 stimulation. TGF-β induced down-regulation of E-cadherin is normally transcriptionally regulated by the Snail-family via SMAD signaling pathway. In accordance with the observation that expression of E-cadherin did not change after TGF-β1-3 treatment of NIH-OVCAR3 the mRNA level of transcription factor Snail was not increased (Figure 5A). Interestingly, significant (p < 0.01) up-regulation of the EMT-related transcription factor ZEB1 mRNA was noted after both 24 h and 72 h (Figure 5B) while no changes was seen in the regulation of another EMT associated transcription factor Twist (data not shown).

### Effect of TGF-β isoforms on tight junctions in NIH-OVCAR3

**Figure 6:**
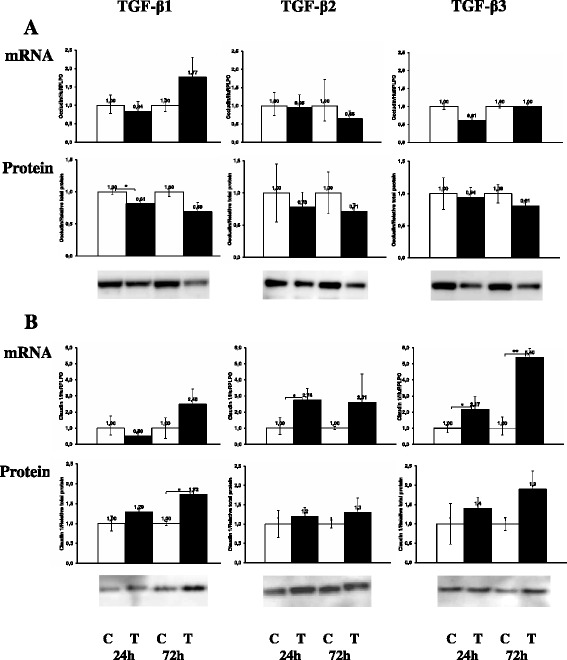
**Effect of TGF-β** **1, TGF-β** **2 or TGF-β** **3 on mRNA and protein expression of tight junction molecules.** There was a significant decrease of occludin protein in TGF-β treated NIH-OVCAR3 cells compared with controls **(A)**, while an increase was noticed of both mRNA and protein expression of claudin 1 in TGF-β treated NIH-OVCAR3 cells compared with controls **(B)**. *P < 0.05.

**Figure 7:**
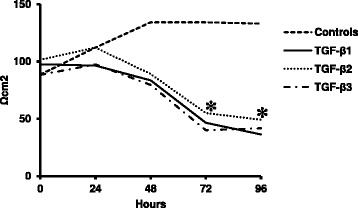
**Effect of TGF-β** **isoforms on transepithelial resistance (TER) in NIH-OVCAR3 cells.** Treatment of TGF-β1, TGF-β2 or TGF-β3 caused a successive decrease in TER after 24 h and reached a significant decrease at 72 h (*P < 0.05). In non-treated cells TER continued to increase to a maximum level of 125 Ωcm^2^ at 48 h and 72 h.

Tight junctions are essential for maintenance of the epithelial phenotype. During EMT, one of the earliest events is the disruption of tight junctions and delocalization of tight junction proteins. In particular claudin-1 is implicated as an active player in EMT and tumor progression. We therefore studied whether TGF-β1, TGF-β2 or TGF-β3 stimulation changed the expression of the tight junction proteins occludin and claudin-1, −3, −4 and −7. In the TGF-β treated NIH-OVCAR3 cells we found significantly (p < 0.05) decreased occludin protein levels but not corresponding changes in mRNA expression (Figure 6A). In contrast, claudin-1 was significantly (p < 0.05) increased by the TGF-β isoforms at both mRNA and protein levels (Figure 6B). No significant changes were found examining the expression of claudin-3, −4 and −7, however there was a tendency for decreased expression of claudin-3 after 72 h TGF-β1-3 treatment. These changes in expression pattern of tight junction proteins were accompanied by loss of the epithelial barrier function in NIH-OVCAR3 cultured on Transwell filters (Figure 7).

## Discussion

Although the role of TGF-β1 in regulating cell proliferation and EMT in ovarian cancers has been studied, little is known about the function of TGF-β2 and TGF-β3 in ovarian tumor progression. Clinical studies have also shown that the three isoforms are overexpressed in ovarian cancers with the predominant expression patterns either dual or triple co-expression [11-13] suggesting that TGF-β1, TGF-β2 and TGF-β3 may function similarly. In this study, we observed that all three TGF-β isoforms stimulated an up-regulation of N-cadherin and migration of NIH-OVCAR3 cells without down regulation of E-cadherin expression or concomitant epithelial to mesenchymal transition. Furthermore, these TGF-β isoforms significantly inhibited ovarian cancer cell growth in a dose-dependent manner, which was in accordance with the inhibitory effect of TGF-β1 on NIH-OVCAR3 cells in previous studies [17,18].

TGF-β may also function as a tumor promoter by inducing EMT along with cell migration and invasion [6,19,20]. Our data suggest that the ovarian cancer cell-line used went into a partial or incomplete EMT [21] with capacity for 2-dimensional migration after TGF-β treatment. During transition from epithelial to mesenchymal phenotype it seems to exist an intermediate phase, expressing two sets of distinct markers [22] not earlier characterized in ovarian cancer. EMT is typically characterized by a functional transition of polarized epithelial cells into mobile mesenchymal cells. The switch of E-cadherin to N-cadherin accompanied with EMT has long been thought as the main reason for the disruption of tight epithelial cell-cell contacts. In the present study we found migrating ovarian cancer cells with unchanged E-cadherin and increased N-cadherin expression after TGF-β treatment and normal epithelial morphology. Recent studies have shown that E-cadherin can be required for intestinal wound healing [23] and that collective migration of colon and squamous cell carcinoma is stabilized and depending on the presence of E-cadherin [21,24]. It is possible that the continuous expression of E-cadherin keeps the cells from entering the mesenchymal phenotype.

Our previous study on normal human ovarian surface epithelium has shown that the expression of Snail, Slug and Zeb1 were increased by TGF-β1 along with a switch from E-cadherin to N-cadherin, decreased occludin expression and induction of complete EMT [7]. Complete EMT has also been described after TGF-β treatment in clearcell ovarian cancer, another ovarian cancer histologic sub-type [5,6,25]. The zinc finger transcription factor Snail, which is a prompt repressor of E-cadherin expression [4], was not affected by TGF-β in OVCAR3. It is likely that E-cadherin escape the expected down regulation due to the lack of Snail expression. However, in our present study TGF-β induced increased expression of Zeb1, also a known repressor of E-cadherin [26,27].

E-cadherin has been well established as a tumor suppressor in a variety of cancer types. Our data adds to the growing evidence that indicates alternative roles for E-cadherin, particularly in ovarian cancers [28]. Unlike other adenocarcinomas such as prostate cancer, E-cadherin expression is increased in late stage EOC where invasion and metastasis is noticed in the whole abdomen and expression of E-cadherin was not correlated to patients’ survival in serous EOC [29,30]. High levels of E-cadherin are ubiquitous expressed in primary ovarian carcinomas, but is low in normal ovarian tissues. E-cadherin is also maintained when ovarian carcinomas metastasize to peritoneum and omentum [30,31]. Previous studies on cultured breast cancer cells have indicated that decreased expression of E-cadherin does not necessarily correlate with invasion [32,33]. Our data supports above clinical findings and suggest that EOC rather spread in the abdomen through collective migration of cancer cells with retained E-cadherin expression then as single cells, reviewed by [21,34].

In the present study we observed coexpression of E-cadherin and N-cadherin in the TGF-β treated ovarian cancer cells without changes of morphological feature, slight decrease of occludin, and reduced transepithelial resistance all indicating dysfunctional tight junctions. Increased claudin-1 could have strengthened the junction but recent studies suggest that claudin-1 can induce an EMT invasive phenotype without alterations in morphology [27]. In this process Claudin-1 also up-regulates Zeb-1 [26,35]. Moreover, N-cadherin can promote invasion and motility even in the presence of E-cadherin in breast cancer cells, suggesting that N-cadherin has a dominant effect over the suggested tumor suppressor functions of E-cadherin [32,33]. Similarly, our study demonstrated the three TGF-β isoforms could induce a significant increase of N-cadherin and claudin-1 expression at both mRNA and protein level and increase migration in the serous adenocarcinoma cell line, NIH-OVCAR3 in the presence of E-cadherin, which suggests the expression of E-cadherin does not preclude the TGF-β induced enhanced migration of NIH-OVCAR3.

Ninety percent of ovarian cancer is of epithelial origin. Still EOC is a very heterogeneous disease comprising of a diverse group of neoplasms exhibiting a wide range morphological characteristics, clinical manifestations, genetic alterations, and tumor behaviors [25,36]. It is histologically sub-grouped into serous, mucinous, endometrioid and clear-cell adenocarinomas. The pato-histology of NIH-OVCAR3 used in the present study is defined as serous ovarian adenocarcinoma, which is the most common type and represents 60% of EOC. In a clear-cell ovarian cancer cell-line all three TGF-β isoforms were capable of inducing complete EMT, E-cadherin repression, mesenchymal transition and invasion [6]. Interestingly, histologic subtype is one main difference between our studies possibly explaining that none of the three TGF-β isoforms could induce complete EMT in NIH-OVCAR3 cells. The initiating events in ovarian cancer development are poorly understood. Recent data based on specific genetic alterations and unique molecular signatures suggest that high-grad serous ovarian cancer could originate from cells within the distal fallopian tube rather than the ovary, while low-grade serous and the other cancer histologic subtypes are thought to arise from ovarian surface epithelium lining the ovary [36]. NIH-OVCAR3, which is originating from a high-grade serous ovarian cancer could theoretically started in the fallopian tube and not in the ovary.

## Conclusion

The present study describes an atypical response to TGF-β and its isoforms 1–3 with regard to migration, which may be described as an incomplete EMT in the most common type of epithelial ovarian cancer. Epithelial ovarian cancer commonly metastasize to the surrounding tissue or inside the peritoneum rather than invade blood vessels to set distance metastasis. Our result raises the question whether ovarian cancer primarily spread via collective migration than via single cell invasion.

## Abbreviations

EMT: Epithelial-to-mesenchymal transition
EOC: Epithelial ovarian cancer
OSE: Ovarian surface epithelium
TGF-β: Transforming growth factor beta
